# Diverse associations between oxidative stress and thromboxane A_2_ in hypertensive glomerular injury

**DOI:** 10.1038/s41440-018-0162-x

**Published:** 2018-12-13

**Authors:** Yukihito Nakano, Yoshihisa Nakatani, Masahiro Takami, Yoshihiro Taniyama, Shuji Arima

**Affiliations:** Division of Nephrology, Department of Internal Medicine, Kindai University Faculty of Medicine, Osaka-Sayama, Japan

**Keywords:** Hypertensive glomerular injury, Albuminuria, Glomerular hypertension, Ozagrel, Tempol

## Abstract

We examined the potential contributions of oxidative stress and thromboxane A_2_ (TXA_2_) to the development of regional heterogeneity in hypertensive glomerular injury using stroke-prone spontaneously hypertensive rats (SHRSP), an animal model of human essential hypertension. We also examined the effect of antioxidant treatment on the regional expression of thromboxane synthase (TXAS) mRNA using a microdissection method. Increases in the glomerular expression of TXAS mRNA were observed in the SHRSP at 15 weeks of age compared with those in the age-matched normotensive control Wistar–Kyoto (WKY) rats: 2.4-fold and 3.1-fold in the superficial and juxtamedullary glomeruli, respectively (*P* < 0.05). The heme oxygenase-1 mRNA expression was markedly increased (greater than eightfold, *P* < 0.05) in both the superficial and juxtamedullary glomeruli in the SHRSP compared with the expression in the WKY rats. In contrast to our expectations, the treatment of SHRSP with tempol (a superoxide dismutase mimetic) significantly (*P* < 0.05) increased the TXAS mRNA expression in the superficial glomeruli and did not improve the histological injury or albuminuria, which were both aggravated. Moreover, ozagrel (a TXAS inhibitor) had a suppressive effect on the TXAS mRNA expression and significantly (*P* < 0.05) improved the histological injury. These results indicated that although TXA_2_ and oxidative stress are linked to each other, TXA_2_ rather than oxidative stress may be a better therapeutic target to improve hypertensive glomerular injury.

## Introduction

Despite recent advances in antihypertensive therapy, hypertension is an important risk factor for the progression of chronic kidney disease and can lead to end-stage kidney disease (ESKD) [1, 2]. Thus, in addition to lowering blood pressure, therapeutic strategies to ameliorate hypertensive renal injury must be established. One potential target is oxidative stress-induced regional ischemia, based on the results of studies that have indicated hypertensive renal injury, which can be inhibited by tempol (a superoxide dismutase mimetic) [3–5], develops early in the juxtamedullary cortex, the renal region most susceptible to ischemic injuries, and subsequently extends toward more superficial regions with an abundant blood supply [6–8].

In addition to high blood pressure, it is well recognized that the renin–angiotensin–aldosterone system (RAAS) and vascular inflammation induce renal oxidative stress under the pathophysiology of hypertension [9, 10]. However, there is currently limited understanding of the other factors involved in the pathophysiology of hypertension. In the present study, we investigated the potential contribution of thromboxane A_2_ (TXA_2_), a vasopressor eicosanoid that frequently contributes to impaired renal hemodynamics under pathological conditions and potently stimulates reactive oxygen species (ROS) generation, to the development of regional heterogeneity in hypertensive glomerular injury [11, 12]. For this purpose, using an animal model of human essential hypertension, we examined the impact of the inhibition of TXA_2_ synthesis in relation to its antioxidant effects on regional glomerular injury. We also determined the regional expression of thromboxane synthase (TXAS) mRNA with and without tempol treatment.

## Methods

### Animals

Experiments were conducted using male stroke-prone spontaneously hypertensive rats (SHRSP/Kpo) and normotensive control Wistar–Kyoto (WKY/Kpo) rats. These rats were originally established in our laboratories (by Drs. Okamoto and Suzuki) [13] and were maintained under controlled conditions of temperature (23 °C ± 2 °C), humidity (50% ± 10%), and a 12-h light/dark cycle (lights on 07.00–19.00 h) in the Central Research Facilities, Kindai University Faculty of Medicine Center for Animal Experiments. The rats were fed standard rat chow and reverse osmotic water ad libitum. All experimental protocols conformed to the guidelines of the National Institutes of Health (Guide for the Care and Use of Laboratory Animals 1966) and were approved by the Institutional Animal Experimentation Committee of Kindai University Faculty of Medicine (KAME-28-013).

### Experimental protocol

#### Protocol 1. Association between glomerular injury and TXAS and heme oxygenase-1 (HO-1) mRNA expression

Rats were housed in a wire-mesh metabolic cage for 24-h urine collection. The systolic blood pressure (SBP) of conscious rats at the ages of 5, 10, and 15 weeks was measured using tail-cuff plethysmography (BP-98A; Softron, Tokyo, Japan). The rats were subsequently anesthetized with an intraperitoneal injection of pentobarbital (Kyoritsuseiyaku Corporation, Tokyo, Japan) (~ 100 mg/kg). Prior to removing their right kidneys, blood was obtained from the abdominal aorta. The kidneys were perfused with phosphate-buffered saline (PBS) at a perfusion pressure that corresponded to the animal’s arterial pressure and were removed. The removed kidneys were subjected to laser microdissection (LMD) to assess the mRNA expression or perform histological analysis.

#### Protocol 2. Pathological roles of TXA_2_ together with oxidative stress

At the age of 10 weeks, the SHRSP were divided into three groups: untreated, treated with tempol at 1 mmol/L, or treated with ozagrel (a TXAS inhibitor) at 3.5 mmol/L. As both tempol and ozagrel were readily soluble in water, they were administered by dissolving the compounds in the drinking water and were provided for 5 weeks. Previous reports have indicated that there was no difference in the blood pressure lowering effect between 1 mmol/L and 3 mmol/L of tempol [14, 15]. We selected tempol at 1 mmol/L because Park et al. [16]. showed that this dose of tempol successfully prevented vascular remodeling and the progression of hypertension in salt-loaded SHRSP at 16 weeks of age. With respect to the dose of ozagrel, Gomi et al. [17] examined the effect of ozagrel with graded doses from 1.4 to 147 mg/kg/day in SHR. They indicated that ozagrel decreases the urinary excretion of thromboxane B_2_ (TXB_2_), which is a stable metabolite of TXA_2_, in a dose-dependent manner. SBP and the production of TXB_2_ from the kidney were decreased only by a high dose of ozagrel. Another researcher reported that 100 mg/kg/day of ozagrel improved proteinuria in streptozotocin-induced diabetic rats [18]. Based on these reports, we decided to use ozagrel at 100 mg/kg/day. We converted the dose of 100 mg/kg/day into 3.5 mmol/L of drinking water because the daily water intake of SHRSP maintained in our laboratories is approximately 30 ml/day. After the 5-week treatment period, the SBP and urinary albumin excretion (UAE) were measured, and the right kidneys were subsequently removed using the same method as previously described.

#### Measurement of oxidative stress and TXB_2_ production

Urine samples were used for the measurement of 8-hydroxy-2′-deoxyguanosine (8-OHdG), which is a marker of oxidative stress and TXB_2_. The urinary excretion of 8-OHdG was determined using a competitive enzyme-linked immunosorbent assay kit (8-OHdG Check; Japan Institute for the Control of Aging, Sizuoka, Japan) [19]. TXB_2_ was measured by an enzyme immuno assay (EIA) kit (R&D systems, Minneapolis, MN, USA) following the instructions of the manufacturer [20].

### Histological analysis

The removed kidneys were divided into two parts at the center of the minor axis. One part was rapidly immersed in OCT compound and immediately frozen in liquid nitrogen for LMD. The other part was fixed with 10% buffered formalin and embedded in paraffin. Subsequently, 4-μm-thick sections were prepared and stained with periodic acid-Schiff (PAS) to assess renal damage. Regional injury was assessed by separately examining the superficial and juxtamedullary glomeruli. The glomerular sclerosis index (GSI) was blindly scored in at least 50 superficial and juxtamedullary glomeruli, in accordance with a previously described method [21]. Each section was scored twice, and the mean score was used for the analysis. The degree of sclerosis was scored as follows: 0, no changes; 1, lesions involving < 25% of the capillary tuft; 2, lesions affecting 25–49% of the capillary tuft; 3, lesions involving 50–75% of the capillary tuft; and 4, lesions involving > 75% of the capillary tuft.

### Immunohistochemical analysis

Sections were deparaffinized, and the antigenic epitopes were retrieved by microwaving in citrate buffer (10 mmol/L, pH 6.0) for 10 min. To inactivate the endogenous peroxidase activity, the sections were subsequently soaked in methanol with 0.3% hydrogen peroxide for 30 min at room temperature. After blocking with 5% goat serum for 30 min, the sections were incubated overnight at 4 °C with the primary antibodies against an oxidative stress marker 8-OHdG (2.5 μg/ml) (Japan Institute for the Control of Aging, Shizuoka, Japan). The sections were washed in PBS, incubated with biotinylated secondary antibody at 1:300 dilution at room temperature for 30 min, incubated with avidin–biotin–peroxidase complex (Vectastain ABC kit; Vector Laboratories) for 30 min and visualized with diaminobenzidine tetrahydrochloride.

### LMD

LMD was performed using the Leica AS LMD system (Leica Microsystems AG, Wetzlar, Germany), in accordance with the manufacturer’s manual [22]. In brief, frozen sections (9–10 μm in thickness) were cut by a cryostat and mounted on a glass slide cover with a 2.5 μm-thick laser pressure-catapulting membrane (PEN foil; Leica Microsystems). The sections were fixed in ethanol:acetic acid (19:1), gently washed with diethylpyrocarbonate (DEPC)-treated water, and stained with 0.05% toluidine blue (TB) solution (pH 7.0, Wako Pure Chemical Industries, Ltd., Osaka, Japan); the TB solution was then rinsed with the DEPC-treated water twice, following which the sections were completely air-dried. The superficial and juxtamedullary glomeruli were separately dissected from the frozen sections using the LMD system and were then immediately collected into a microcentrifuge tube cap filled with lysis buffer.

### mRNA level determination

Total RNA from the microdissected glomeruli was extracted using an RNeasy mini kit (Qiagen, Valencia, CA). Isolated RNA samples were reverse-transcribed with a high-capacity RNA-to-cDNA kit (Applied Biosystems, Foster City, CA), and the TaqMan real-time PCR assay (Applied Biosystem) was employed for the following molecules: TXAS (Rn00562160_m1) and HO-1 (Rn00561387_m1). Amplification data were analyzed using Sequence Detection Software (SDS ver. 1.9), and the mRNA levels of each target were normalized to the level of β-actin using the ΔΔCt comparative method [23].

### Mean glomerular volume (MGV) measurement

The MGV was estimated using the 2-profile method [24]. In brief, kidney specimens were sectioned at 5 µm thickness and stained with PAS. Two sections were made at 20-µm intervals onto sequential slides. At least 10 individual nonsclerotic glomeruli (sclerotic lesions < 75%) were randomly selected from both the superficial and juxtamedullary cortices. Glomerular images were obtained using a color video camera attached to a microscope (Nikon Instruments Inc., Melville, NY) at × 200 magnification. In each captured image, the glomerular tuft was digitally traced, and the areas were calculated using computer image analysis software (NIS-Elements D 3.22; Nikon Instruments Inc., Melville, NY). The MGV was subsequently calculated based on the areas of the two sections. We can obtain almost the same results using the 2-profile method compared with the Cavalieri method [25], which is the most reliable and accurate method to estimate the MGV if more than eight glomeruli are analyzed.

### Statistical analysis

All values are expressed as the mean ± SEM and were analyzed using unpaired Student’s *t* test and one-way analysis of variance. *P* values < 0.05 were considered statistically significant.

## Results

### Association between glomerular injury and TXAS and HO-1 mRNA expression

**Fig. 1:**
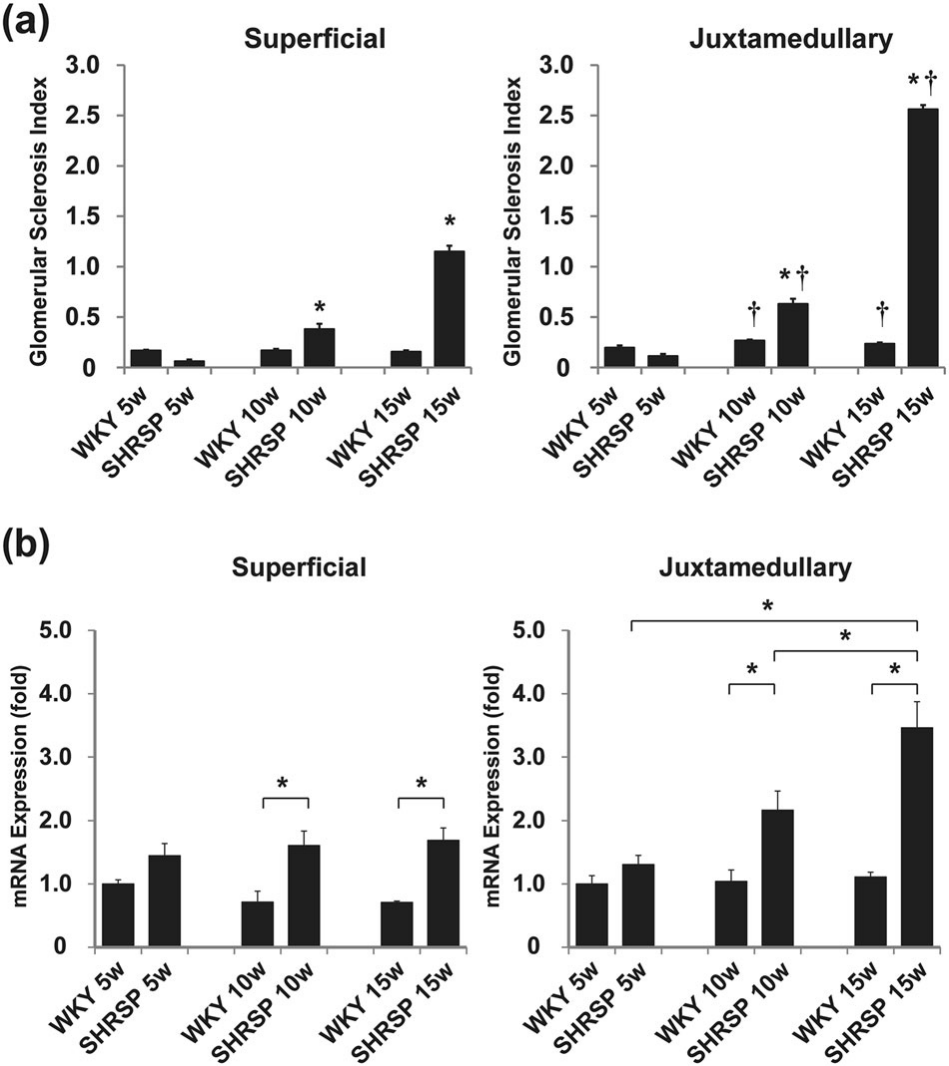
Histological analysis and TXAS mRNA expression in superficial and juxtamedullary glomeruli. **a** GSI of superficial and juxtamedullary glomeruli in WKY rats and SHRSP at 5, 10, and 15 weeks of age. **b** Comparison of TXAS mRNA expression between WKY rats and SHRSP in superficial and juxtamedullary glomeruli. *P < 0.05 vs. WKY rats at the same age, ^†^*P* < 0.05 vs. superficial glomeruli at the same age

**Fig. 2:**
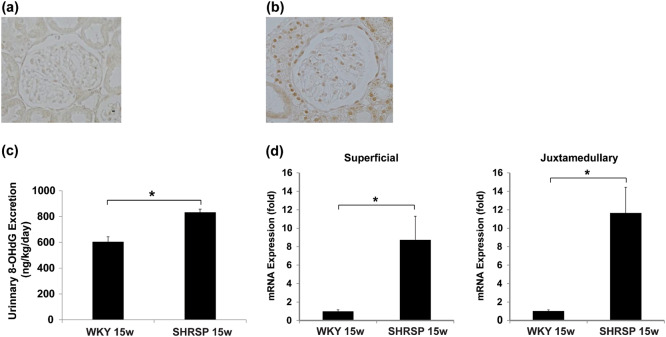
Oxidative stress in the kidneys. **a**, **b** Immunohistochemical staining for 8-OHdG in WKY rats **a** and SHRSP **b** at 15 weeks of age. Original magnification × 100. **c** Urinary 8-OHdG excretion in WKY rats and SHRSP at 15 weeks of age. **d** Comparison of the HO-1 mRNA expression between WKY rats and SHRSP in superficial and juxtamedullary glomeruli at 15 weeks of age. *P < 0.05

The characteristics of the WKY rats and SHRSP are shown in Table 1. The body weight of the SHRSP was significantly (*P* < 0.05) less at the ages of 10 and 15 weeks than that of the WKY rats. In the SHRSP, the SBP rapidly increased as the rats grew and was significantly (*P* < 0.05) high during the entire experimental period compared with that in the WKY rats. Although the renal function, estimated as the serum creatinine concentration (SCr) and creatinine clearance (CCr)/100-g body weight (BW), did not change during the period in the WKY rats, it significantly declined in the SHRSP at 15 weeks of age. The UAE progressively increased only in the SHRSP. The urinary TXB_2_ excretion was significantly greater in the SHRSP than in the WKY rats (22.6 ± 2.5 ng/day vs. 11.3 ± 1.0 ng/day, respectively, *P* < 0.05). Light microscopy showed high GSI in the SHRSP at ≥ 10 weeks of age. In addition, when we estimated each rat’s glomerular injury, a substantially higher GSI was observed in the juxtamedullary glomeruli than in the superficial glomeruli (Fig. 1a). The TXAS mRNA expression was enhanced in both the superficial and juxtamedullary glomeruli in the SHRSP compared with that in the age-matched WKY rats (Fig. 1b). In the superficial glomeruli, this increase nearly doubled at 10 weeks of age and remained at this level until 15 weeks of age. Moreover, in the juxtamedullary glomeruli, a progressive enhancement occurred and became 3.1-fold at 15 weeks of age. Immunohistochemical analysis indicated greater numbers of 8-OHdG-positive cells in both the glomerulus and tubules in the 15-week-old SHRSP than in the age-matched WKY rats (Fig. 2a and b). Moreover, as expected, the urinary 8-OHdG excretion was significantly greater in the SHRSP than in the WKY rats (830.7 ± 26.6 ng/kg/day vs. 602.3 ± 40.9 ng/kg/day; Fig. 2c). The HO-1 mRNA expression was also markedly increased in both the superficial and juxtamedullary glomeruli (8.7-fold and 11.6-fold, respectively) in the SHRSP at 15 weeks of age compared with that in the age-matched WKY rats (Fig. 2d), although the difference was greater in the juxtamedullary glomeruli than in the superficial glomeruli.

**Table 1:** The characteristics of WKY rats and SHRSP at 5, 10, and 15 weeks of age

	WKY rats			SHRSP		
	5 weeks	10 weeks	15 weeks	5 weeks	10 weeks	15 weeks
	(*n* = 6)	(*n* = 6)	(*n* = 6)	(*n* = 6)	(*n* = 7)	(*n* = 8)
BW (g)	113 ± 2	321 ± 8	399 ± 5	109 ± 1	266 ± 4^*^	274 ± 13^*^
SBP (mmHg)	134.6 ± 4.6	146.7 ± 2.7	140.0 ± 2.0	147.9 ± 2.9^*^	238.4 ± 6.6^*^	249.9 ± 5.1^*^
Serum Cr (mg/dl)	0.20 ± 0.00	0.26 ± 0.00	0.30 ± 0.01	0.16 ± 0.01 ^*^	0.24 ± 0.01	0.41 ± 0.03^*†^
CCr/100-g BW (ml/min/100-g BW)	0.69 ± 0.02	0.71 ± 0.04	0.68 ± 0.04	0.74 ± 0.02	0.74 ± 0.03	0.53 ± 0.06^*†^
UAE (mg/day)	0.02 ± 0.00	0.12 ± 0.03	0.05 ± 0.01^†^	0.01 ± 0.00	0.21 ± 0.04^*^	1.73 ± 0.17^*†^

*BW* body weight, *SBP* systolic blood pressure, *Cr* creatinine, *CCr* creatinine clearance, *UAE* urinary albumin excretion

Data are indicated as mean ± SEM

^*^P < 0.05 vs. WKY rats at the same age

^†^*P* < 0.05 vs. the same species at 10 weeks of age

### Pathological roles of TXA_2_ together with oxidative stress

**Fig. 3:**
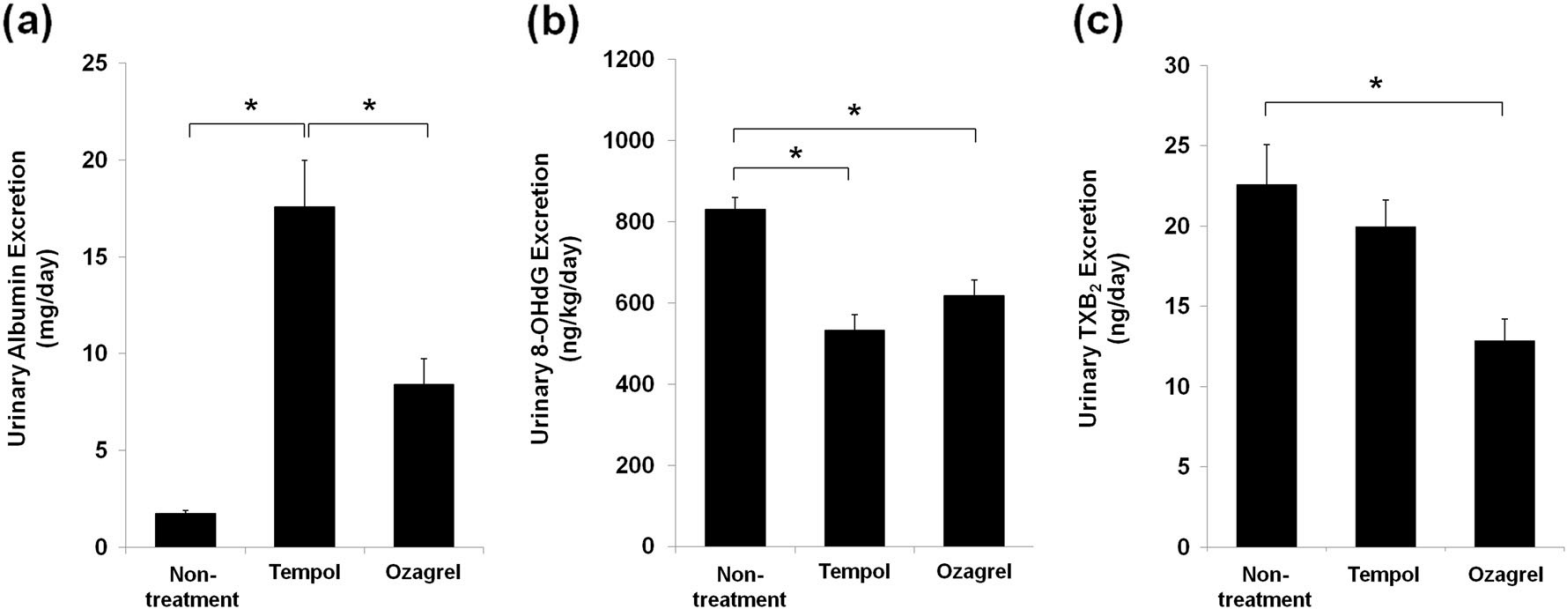
Effects of tempol or ozagrel administration on urinary albumin **a**, 8-OHdG **b**, and TXB_2_ excretion **c**. *P < 0.05

**Fig. 4:**
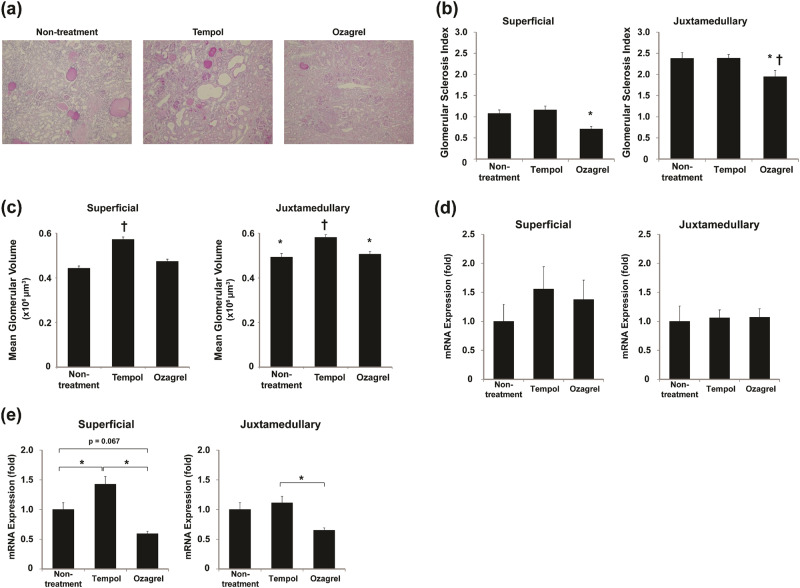
Effects of tempol or ozagrel on histological injury and HO-1 and TXAS mRNA expression. **a** Representative photomicrographs of renal cortex stained with PAS. Original magnification × 100. **b** Glomerular injury estimated by GSI after tempol and ozagrel treatments. Original magnification × 100. *P < 0.05 vs. untreated group, ^†^*P* < 0.05 vs. tempol-treated group. **c** Morphometric analysis using the two-profile method (described in the Methods). *P < 0.05 vs. superficial glomeruli, ^†^*P* < 0.05 vs. untreated group. **d**, **e** Comparisons of HO-1 **d** and TXAS **e** mRNA expression between untreated SHRSP and tempol- or ozagrel-treated SHRSP in superficial and juxtamedullary glomeruli. *P < 0.05

Tempol or ozagrel did not have an effect on the BW, SBP, SCr, or CCr/100 g BW in the SHRSP (Table 2). In contrast, both tempol and ozagrel exacerbated the UAE (tempol: 17.4 ± 0.2 mg/day, ozagrel: 8.4 ± 1.3 mg/day; Fig. 3a), although they reduced the urinary 8-OHdG excretion (tempol: 532.3 ± 38.9 ng/kg/day, ozagrel: 616.8 ± 39.2 ng/kg/day; Fig. 3b). Ozagrel significantly reduced the urinary TXB_2_ excretion, whereas tempol did not induce changes (tempol: 20.0 ± 1.7 ng/day, ozagrel: 12.9 ± 1.3 ng/day; Fig. 3c). The exacerbation of the UAE was substantially stronger in the tempol-treated rats than in the ozagrel-treated rats. In addition, ozagrel, but not tempol, improved the histological lesions (Fig. 4a) and GSI in both the superficial and juxtamedullary glomeruli (Fig. 4b). Quantitative morphometric analysis of the glomeruli showed that the MGV of the superficial or juxtamedullary glomeruli was 29% or 18% greater, respectively, in the tempol-treated group than in the untreated group. In contrast, no difference was observed in the ozagrel-treated group in the superficial or juxtamedullary glomeruli (Fig. 4c). The treatments did not have a significant effect on the HO-1 mRNA expression in the superficial or juxtamedullary glomeruli (Fig. 4d), although both treatments reduced the urinary 8-OHdG excretion, as previously discussed (Fig. 3b). The TXAS mRNA expression is shown in Fig. 4e. Tempol significantly increased the TXAS mRNA expression in the superficial glomeruli (1.4-fold), but not in the juxtamedullary glomeruli, whereas ozagrel suppressed it. The TXAS mRNA expression was significantly less in both the superficial and juxtamedullary glomeruli (0.7-fold and 0.7-fold, respectively) in the ozagrel-treated rats compared with that in the tempol-treated rats.

**Table 2:** Changes in parameters after treatment

	Untreated	Tempol	Ozagrel
	(*n* = 8)	(*n* = 8)	(*n* = 7)
BW (g)	274 ± 13	282 ± 12	262 ± 9
SBP (mmHg)	249.9 ± 5.1	259.2 ± 5.6	252.4 ± 4.9
Serum Cr (mg/dl)	0.41 ± 0.03	0.43 ± 0.2	0.40 ± 0.01
CCr/100-g BW (ml/min/100-g BW)	0.53 ± 0.06	0.51 ± 0.02	0.50 ± 0.01

*BW* body weight, *SBP* systolic blood pressure, *Cr* creatinine, *CCr* creatinine clearance

Data are indicated as mean ± SEM

No significant difference was found in any parameters among groups

## Discussion

Studies have demonstrated that hypertensive glomerular sclerosis develops early in the juxtamedullary cortex and subsequently extends toward the superficial cortex in rats and humans [6–8]. Although it has been proposed that differences in the perfusion pressure or oxidative states between the juxtamedullary and superficial glomeruli are responsible for this regional heterogeneity, the precise mechanisms that underlie this effect are unclear. Thus, in the present study, using an animal model of severe essential hypertension, we examined the potential involvements of TXA_2_ together with oxidative stress in the pathophysiology of hypertensive glomerular injury. We confirmed this heterogeneity and identified increased oxidative stress together with preferentially enhanced TXAS mRNA expression in the juxtamedullary glomeruli in SHRSP. To our knowledge, this investigation is the first report to demonstrate the potential involvement of TXA_2_ in regional heterogeneity in hypertensive glomerular injury. We also found that TXA_2_ production can be affected by antioxidant therapy and may affect the degree of UAE.

The importance of oxidative stress in the pathogenesis of hypertensive organ damage has been extensively studied [16, 26–29]. The excessive production or decreased metabolism of ROS, such as superoxide anion (O_2_^−^), hydrogen peroxide (H_2_O_2_), and hydroxyl anion (OH^−^), can lead to oxidative stress that alters the redox state and causes the redirection of redox-regulated signaling pathways and cellular dysfunction or damage [30]. It is well recognized that RAAS and vascular inflammation induce renal oxidative stress [9, 10]. However, antihypertensive treatment with RAAS blockers may not be completely effective at preventing the progression of hypertensive renal injury to ESKD. Thus, for better antihypertensive treatment, there is a need to determine other factors involved in the pathogenesis of hypertensive glomerular injury. In the present study, we investigated the potential involvement of TXA_2_ in the pathophysiology of hypertensive glomerular injury as it has been reported that the renal synthesis of TXA_2_ increases in SHRSP [31]. TXA_2_ is an arachidonic acid metabolite converted by TXAS, which by acting on its receptor [thromboxane prostanoid receptor (TPR)] causes platelet aggregation, the contraction of vascular smooth muscle cells and increases the expression of adhesion molecules in endothelial cells [32, 33]. In the kidneys, TXAS and TPR are expressed in endothelial cells and glomerular mesangial cells, and TXA_2_ decreases the glomerular filtration rate (GFR) under pathological conditions [34–36]. Gelosa et al. [37] have reported that terutroban, a selective TPR antagonist, prevented vascular hypertrophy and improved the development of proteinuria without affecting blood pressure in SHRSP. These findings imply that the TXA2–TPR pathway mediates glomerular injury through a blood pressure-independent mechanism in severe hypertension. Interestingly, the TXA_2_–TPR pathway and oxidative stress form a vicious circle, i.e., TXA_2_ stimulates the generation of ROS and O_2_^−^ [11, 12], which, in turn, stimulate TXA_2_ synthesis by TXAS upregulation [38]. This background prompted us to examine their potential associations with the pathogenesis of hypertensive glomerular injury, which we identified in diverse forms.

We found that both the TXAS mRNA expression and oxidative stress increased in SHRSP particularly in the juxtamedullary glomeruli, which showed more severe hypertensive injury than that in the superficial glomeruli. These findings support the idea that TXA_2_ together with oxidative stress contribute to the development of hypertensive glomerular injury. However, tempol showed a tendency to cause marked increases in the UAE and significantly reduced renal oxidative stress as estimated by the urinary 8-OHdG excretion (Fig. 3b), although it may reflect interstitial and glomerular oxidative stress. These results are completely consistent with those of Sugama et al. [39], who reported that tempol aggravated renal injury in advanced-stage SHRSP. In addition, clinical and experimental studies have reported conflicting results with antioxidant therapies [40, 41]. Moreover, the increase in the UAE was substantially smaller when TXAS was inhibited with ozagrel, although it reduced renal oxidative stress to an extent similar to that by tempol. Thus, it is possible that antioxidant therapy through TXA_2_ inhibition is a better therapeutic target than ROS inhibition to inhibit the aggravation of hypertensive glomerular injury at an advanced stage.

The reason why tempol exacerbated UAE remains unclear. Sugama et al. [39] hypothesized that tempol aggravates renal injury in advanced-stage hypertension by inducing glomerular hypertension in residual nephrons (mostly superficial nephrons) through an inadequate increase in regional blood flow or an attenuation of the tubuloglomerular feedback (TGF) response. It has been reported that increased O_2_^−^ production in SHR enhances the TGF response, which is blunted by tempol, leading to the development of glomerular hypertension [42]. It has also been demonstrated that increased mechanical stress on glomeruli, such as glomerular hypertension, upregulates TXAS mRNA, which results in increased TXA_2_ production and a further increase in the glomerular capillary pressure [43]. These findings may be the mechanism by which tempol upregulates TXAS mRNA. Consistent with this hypothesis, we found that tempol but not ozagrel increased the MGV in both the superficial and juxtamedullary cortices (particularly the superficial cortex) without improving histological lesions and GSI. Thus, tempol may exacerbate UAE by increasing the glomerular capillary pressure, whereas ozagrel does not cause glomerular hypertension. In addition to this possibility, TXAS upregulation induced by tempol (particularly in superficial glomeruli) may be responsible for the exacerbation of the UAE because TXA_2_ is known to accelerate glomerular injury through the activation of adhesion molecules or the stimulation of mesangial cell proliferation under pathological conditions [32, 44, 45]. However, this possibility is not consistent with our finding that ozagrel significantly (but substantially less than tempol) increased the UAE. Because ozagrel does not increase the glomerular capillary pressure or cause glomerular histological injury (as previously discussed), the inhibition of TXA_2_ synthesis with ozagrel may increase the UAE via the elevation of the glomerular ultrafiltration coefficient under conditions with advanced hypertensive glomerular injury. Consistent with this concept, TXA_2_ is known to decrease the ultrafiltration coefficient and GFR [44]. Alternatively, ozagrel might influence the metabolism of other arachidonates, which may lead to the exacerbation of UAE. Although the reason ozagrel did not increase the CCr/BW remains unclear, it may be that an increased glomerular ultrafiltration coefficient could compensate but not surpass the remaining severe glomerular damage. Further studies exploring the precise pathological roles and underlying mechanisms of the involvement of the TXA_2_–TPR pathway in the pathophysiology of hypertensive renal injury are clearly required.

Our study has several limitations. First, although we selected the concentration of tempol and ozagrel based on the reasons described, the dose-dependent effects of these drugs must be elucidated. Second, the potential involvement of other prostanoids, such as prostacyclin, in hypertensive glomerular injury must also be verified.

In conclusion, the TXA_2_–TPR pathway and oxidative stress participate and interact together to promote hypertensive glomerular injury. Our results indicated that antioxidant therapy through TXA_2_ inhibition may be a better therapeutic target than ROS inhibition to inhibit the aggravation of hypertensive glomerular injury at an advanced stage.

## References

[1] Siewert-Delle A, Ljungman S, Andersson OK, Wilhelmsen L (1998). Does treated primary hypertension lead to end-stage renal disease? A 20-year follow-up of the Primary Prevention Study in Göteborg, Sweden. Nephrol Dial Transplant.

[2] Sarnak MJ, Greene T, Wang X, Beck G, Kusek JW, Collins AJ (2005). The effect of a lower target blood pressure on the progression of kidney disease: long-term follow-up of the modification of diet in renal disease study. Ann Intern Med.

[3] Hisaki R, Fujita H, Saito F, Kushiro T (2005). Tempol attenuates the development of hypertensive renal injury in Dahl salt-sensitive rats. Am J Hypertens.

[4] Nishiyama A, Yao L, Nagai Y, Miyata K, Yoshizumi M, Kagami S (2004). Possible contributions of reactive oxygen species and mitogen-activated protein kinase to renal injury in aldosterone/salt-induced hypertensive rats. Hypertension.

[5] Nishiyama A, Yoshizumi M, Hitomi H, Kagami S, Kondo S, Miyatake A (2004). The SOD mimetic tempol ameliorates glomerular injury and reduces mitogen-activated protein kinase activity in Dahl salt-sensitive rats. J Am Soc Nephrol.

[6] Bohle A, Biwer E, Christensen JA (1988). Hyperperfusion injury of the human kidney in different glomerular diseases. Am J Nephrol.

[7] Iversen BM, Amann K, Kvam FI, Wang X, Ofstad J (1998). Increased glomerular capillary pressure and size mediate glomerulosclerosis in SHR juxtamedullary cortex. Am J Physiol.

[8] Mori T, Cowley AW (2004). Role of pressure in angiotensin II-induced renal injury: chronic servo-control of renal perfusion pressure in rats. Hypertension.

[9] Higuchi S, Ohtsu H, Suzuki H, Shirai H, Frank GD, Eguchi S (2007). Angiotensin II signal transduction through the AT1 receptor: novel insights into mechanisms and pathophysiology. Clin Sci (Lond).

[10] Yao EH, Fukuda N, Matsumoto T, Kobayashi N, Katakawa M, Yamamoto C (2007). Losartan improves the impaired function of endothelial progenitor cells in hypertension via an antioxidant effect. Hypertens Res.

[11] Wang C, Luo Z, Kohan D, Wellstein A, Jose PA, Welch WJ (2015). Thromboxane prostanoid receptors enhance contractions, endothelin-1 and oxidative stress in microvessels from mice with CKD. Hypertension.

[12] Ellinsworth DC, Shukla N, Fleming I, Jeremy JY (2014). Interactions between thromboxane A_2_, thromboxane/prostaglandin (TP) receptors, and endothelium-derived hyperpolarization. Cardiovasc Res.

[13] Okamoto K, Yamamoto K, Morita N, Ohta Y, Chikugo T, Higashizawa T (1986). Establishment and use of the M strain of stroke-prone spontaneously hypertensive rat. J Hypertens.

[14] Simonsen U, Christensen FH, Buus NH (2009). The effect of tempol on endothelium-dependent vasodilatation and blood pressure. Pharmacol Ther.

[15] Dornas WC, Silva M, Tavares R, de Lima WG, dos Santos RC, Pedrosa ML (2015). Efficacy of the superoxide dismutase mimetic tempol in animal hypertension models: a meta-analysis. J Hypertens.

[16] Park JB, Touyz RM, Chen X, Schiffrin EL (2002). Chronic treatment with a superoxide dismutase mimetic prevents vascular remodeling and progression of hypertension in salt-loaded stroke-prone spontaneously hypertensive rats. Am J Hypertens.

[17] Gomi T, Ikeda T, Ishimitsu T, Uehara Y (1989). Protective effect of thromboxane synthetase inhibitor on hypertensive renal damage in Dahl salt-sensitive rats. Prostaglandins Leukot Essent Fat Acids.

[18] Hora K, Oguchi H, Furukawa T, Hora K, Tokunaga S (1990). Effects of a selective thromboxane synthetase inhibitor OKY-046 on experimental diabetic nephropathy. Nephron.

[19] Negishi H, Njelekela M, Ikeda K, Sagara M, Noguchi T, Kuga S (2000). Assessment of in vivo oxidative stress in hypertensive rats and hypertensive subjects in Tanzania, Africa. Hypertens Res.

[20] Pidgeon GP, Tamosiuniene R, Chen G, Leonard I, Belton O, Bradford A (2004). Intravascular thrombosis after hypoxia-induced pulmonary hypertension: regulation by cyclooxygenase-2. Circulation.

[21] Raij L, Azar S, Keane W (1984). Mesangial immune injury, hypertension, and progressive glomerular damage in Dahl rats. Kidney Int.

[22] Kobayashi T, Uehara S, Ikeda T, Itadani H, Kotani H (2003). Vitamin D_3_ up-regulated protein-1 regulates collagen expression in mesangial cells. Kidney Int.

[23] Livak KJ, Schmittgen TD (2001). Analysis of relative gene expression data using real-time quantitative PCR and the 2 (-Delta C(T)) Method. Methods.

[24] Najafian B, Basgen JM, Mauer M (2002). Estimating mean glomerular volume using two arbitrary parallel sections. J Am Soc Nephrol.

[25] Gundersen HJ, Jensen EB (1987). The efficiency of systematic sampling in stereology and its prediction. J Microsc.

[26] Niazi ZR, Silva GC, Ribeiro TP, León-González AJ, Kassem M, Mirajkar A (2017). EPA:DHA 6:1 prevents angiotensin II-induced hypertension and endothelial dysfunction in rats: role of NADPH oxidase- and COX-derived oxidative stress. Hypertens Res.

[27] Kitiyakara C, Chabrashvili T, Chen Y, Blau J, Karber A, Aslam S (2003). Salt intake, oxidative stress, and renal expression of NADPH oxidase and superoxide dismutase. J Am Soc Nephrol.

[28] Tanito M, Nakamura H, Kwon YW, Teratani A, Masutani H, Shioji K (2004). Enhanced oxidative stress and impaired thioredoxin expression in spontaneously hypertensive rats. Antioxid Redox Signal.

[29] Wilcox CS (2005). Oxidative stress and nitric oxide deficiency in the kidney: a critical link to hypertension?. Am J Physiol.

[30] Wilcox CS (2002). Reactive oxygen species: roles in blood pressure and kidney function. Curr Hypertens Rep.

[31] Kawaguchi H, Saito H, Yasuda H (1987). Renal prostaglandins and phospholipase A_2_ in spontaneously hypertensive rats. J Hypertens.

[32] Ishizuka T, Suzuki K, Kawakami M, Hidaka T, Matsuki Y, Nakamura H (1996). Thromboxane A_2_ receptor blockade suppresses intercellular adhesion molecule-1 expression by stimulated vascular endothelial cells. Eur J Pharmacol.

[33] Davì G, Patrono C (2007). Platelet activation and atherothrombosis. N Engl J Med.

[34] Spurney RF, Fan PY, Ruiz P, Sanfilippo F, Pisetsky DS, Coffman TM (1992). Thromboxane receptor blockade reduces renal injury in murine lupus nephritis. Kidney Int.

[35] Abe T, Takeuchi K, Takahashi N, Tsutsumi E, Taniyama Y, Abe K (1995). Rat kidney thromboxane receptor: molecular cloning, signal transduction and intrarenal expression localization. J Clin Invest.

[36] Kobayashi T, Suzuki J, Watanabe M, Suzuki S, Yoshida K, Kume K (1997). Changes in platelet calcium concentration by thromboxane A_2_ stimulation in patients with nephrotic syndrome of childhood. Nephron.

[37] Gelosa P, Sevin G, Pignieri A, Budelli S, Castiglioni L, Blanc-Guillemaud V (2011). Terutroban, a thromboxane/prostaglandin endoperoxide receptor antagonist, prevents hypertensive vascular hypertrophy and fibrosis. Am J Physiol Heart Circ Physiol.

[38] Korbecki J, Baranowska Bosiacka I, Gutowska I, Chlubek D (2013). The effect of reactive oxygen species on the synthesis of prostanoids from arachidonic acid. J Physiol Pharmacol.

[39] Sugama I, Kohagura K, Yamazato M, Nakamura T, Shinzato T, Ohya Y (2014). Superoxide dismutase mimetic, tempol, aggravates renal injury in advanced-stage stroke-prone spontaneously hypertensive rats. J Hypertens.

[40] Jialal I, Devaraj S (2003). Antioxidants and atherosclerosis: don’t throw out the baby with the bath water. Circulation.

[41] Bjelakovic G, Nikolova D, Gluud LL, Simonetti RG, Gluud C (2012). Antioxidant supplements for prevention of mortality in healthy participants and patients with various diseases. Cochrane Database Syst Rev.

[42] Wilcox CS (2003). Redox regulation of the afferent arteriole and tubuloglomerular feedback. Acta Physiol Scand.

[43] Anwar MA, Shalhoub J, Lim CS, Gohel MS, Davies AH (2012). The effect of pressure-induced mechanical stretch on vascular wall differential gene expression. J Vasc Res.

[44] Remuzzi G, FitzGerald GA, Patrono C (1992). Thromboxane synthesis and action within the kidney. Kidney Int.

[45] Spurney RF, Onorato JJ, Albers FJ, Coffman TM (1993). Thromboxane binding and signal transduction in rat glomerular mesangial cells. Am J Physiol.

